# B Cell Reconstitution and Influencing Factors After Hematopoietic Stem Cell Transplantation in Children

**DOI:** 10.3389/fimmu.2019.00782

**Published:** 2019-04-12

**Authors:** Nicolaas G. van der Maas, Dagmar Berghuis, Mirjam van der Burg, Arjan C. Lankester

**Affiliations:** Willem-Alexander Children's Hospital, Department of Pediatrics and Laboratory for Pediatric Immunology, Leiden University Medical Center, Leiden, Netherlands

**Keywords:** hematopoietic stem cell transplantation, allogeneic, immune reconstitution, B lymphocyte, subsets, pediatric

## Abstract

B cell reconstitution after hematopoietic stem cell transplantation (HSCT) is variable and influenced by different patient, donor, and treatment related factors. In this review we describe B cell reconstitution after pediatric allogeneic HST, including the kinetics of reconstitution of the different B cell subsets and the development of the B cell repertoire, and discuss the influencing factors. Observational studies show important roles for stem cell source, conditioning regimen, and graft vs. host disease in B cell reconstitution. In addition, B cell recovery can play an important role in post-transplant infections and vaccine responses to encapsulated bacteria, such as pneumococcus. A substantial number of patients experience impaired B cell function and/or dependency on Ig substitution after allogeneic HSCT. The underlying mechanisms are largely unresolved. The integrated aspects of B cell recovery after HSCT, especially BCR repertoire reconstitution, are awaiting further investigation using modern techniques in order to gain more insight into B cell reconstitution and to develop strategies to improve humoral immunity after allogeneic HSCT.

## Introduction

Hematopoietic stem cell transplantation (HSCT) is a treatment modality in which hematopoietic stem cells are used as curative therapy for congenital and acquired disorders of the hematopoietic system and metabolic diseases (1). During HSCT, the hematopoietic system is replaced using donor-derived hematopoietic stem cells as allograft. Stem cells can give progeny to functioning erythrocytes, thrombocytes, myeloid lineages and/or lymphocytes, achieving recovery of normal hematopoiesis and immunity.

Restoration of the individual components of the immune system occurs with different dynamics in which innate immunity (neutrophils, monocytes and natural killer cells) typically precedes adaptive immunity (T- and B-lymphocytes). Complete immune reconstitution can take several months up to 2 years after HSCT. Immune reconstitution after allogeneic HSCT has been studied extensively with a main focus on T cell reconstitution. Only limited information is available about B cell reconstitution. In this review we summarize the existing knowledge on B cell reconstitution after pediatric allogeneic HSCT and point out the need and challenges for further investigations. We included studies via systematic literature search in Embase, Medline Epub (Ovid), Cochrane Central and Web of Science including the terms “hematopoietic stem cell transplantation,” “B lymphocyte,” “immune reconstitution,” “child” and synonyms between 01-01-1980 and 31-12-2018. Additional relevant studies were included through references within the identified studies. Lastly, top results of Google Scholar were screened.

## B Cell Reconstitution After HSCT

**Figure 1:**
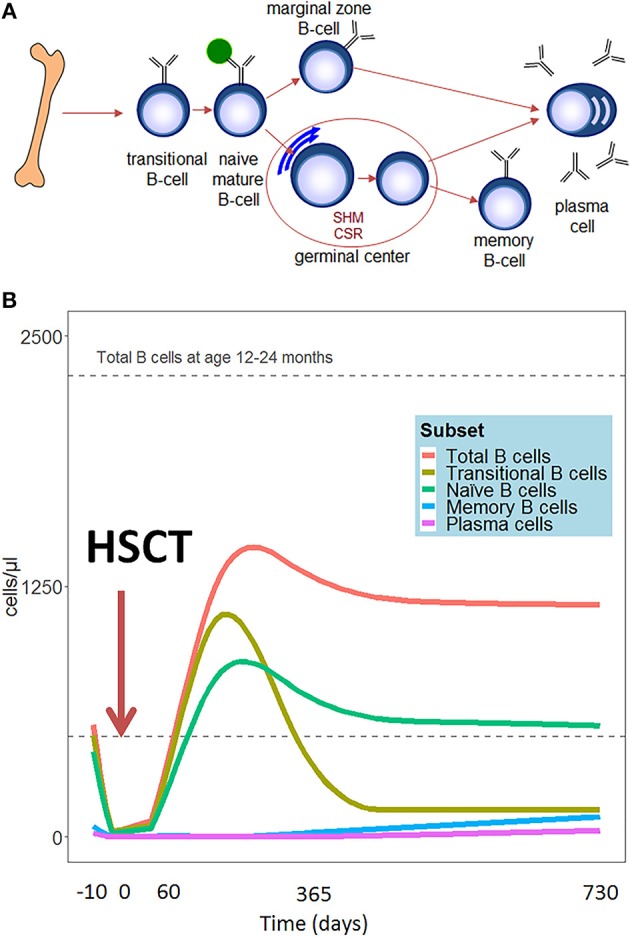
**(A)** Schematic representation of peripheral B-cell development. **(B)** Hypothetical scheme of B cell subset reconstitution after HSCT based on literature. The first cells emerging in the peripheral blood are the transitional B cells. In the course of the first year, the transitional B cells decrease in number and are replaced by mature naïve B cells. These mature B lymphocytes further differentiate into memory B cells and plasma cells.

Compared to other hematopoietic cell types, B cell reconstitution occurs relatively late after HSCT. The first emerging B cells can be detected in peripheral blood after 1.5–2 months. These are mainly CD24high CD38high transitional B cells (2) (See Figure 1A for all peripheral B-cell subsets). A subdivision of these transitional B cells can be made in the early appearing CD21low B cells, called the T1 cells, and the CD21high B (or T2) cells which develop later (3). Additionally, expansion of CD5+ B cells is reported early after HSCT (4, 5). CD5 is a known marker of human immature B cells, but its expression covers a broader range of B cell developmental/differentiation stages. More mature CD5+ B cell subpopulations are characterized by their regulatory properties such as IL-10 production upon activation by bacterial or parasitic antigens, and their constitutive death-inducing ligand expression (6–10). The CD5+ B cell expansion early after HSCT is likely to be a reflection of B cell immaturity, but may also point to a role for regulatory B cells in controlling immune reactions and autoimmunity after HSCT. Several months after HSCT, the transitional B cells decrease in number and gradually naive mature (CD24int CD38int) B cells emerge to become the predominant population. In the course of the first year following HSCT, naive mature B cells represent more than 80% of the peripheral blood B lymphocytes. (2). To obtain more insight in the mechanism and dynamics of B cell regeneration, κ-deleting recombination excision circles (KRECs) might serve as a useful biomarker for replication history and to evaluate the onset of *de novo* B-cells, as KRECs have been reported to be positively correlated with B cell numbers after transplantation (11–15). At one year after HSCT, the B cell reconstitution stabilizes reaching age-corrected normal total B cell counts in peripheral blood in most patients (Figure 1B) (16–20). Looking further into the B cell populations, non-switched (CD27+IgM+IgD+/-) and switched (CD27+IgD-IgM-) memory B-cells appear slowly, taking up to two years or longer after HSCT to reach normal age matched levels (16, 17, 20, 21). Especially non-switched B cells seem to remain below normal values, suggesting defects in this maturation stage. During the process of B cell maturation in general, mature B lymphocytes further differentiate into memory B cells, and may undergo isotype switching and affinity maturation in a T cell dependent germinal center reaction (Figure 1A). In this process, cognate interaction between T follicular helper (Tfh) cells and specialized follicular dendritic cells is pivotal. As a consequence, the quality and dynamics of CD4 T cell, and thus also Tfh, reconstitution after HSCT will also impact on B cell differentiation and may thus contribute to an impaired or arrested maturation of B cells. (22–24). However, even in the presence of donor CD4+ T cells that are capable of supporting the process of somatic hyper mutation, the incidence of somatic hypermutation is decreased in recipient B cells in cell culture (25). It could be that treatment given prior to transplantation disrupts secondary lymphoid organs, which are necessary for the introduction of somatic hypermutations in the variable domains of the immunoglobulin molecules and affinity maturation in the germinal centers (26). Immune responses against polysaccharides seem frequently impaired in HSCT patients (27, 28). Polysaccharide antibody responses are important for the T cell independent defense to encapsulated bacteria, in which marginal zone B cells play an important role (29, 30). The impaired reconstitution of this subset might indicate why certain patients encounter specific problems with susceptibility to encapsulated bacteria such as pneumococcus. The counterpart of marginal zone B cells, IgM memory B cells, seems also to be reduced in long term transplanted patients (16, 17, 20, 21).

Immunoglobulin (Ig) levels seem to recover in parallel to B cell reconstitution, in which recovery of Ig subclasses usually occurs in a distinctive order (16, 31–33). After HSCT, Ig levels drop, reflecting the absence of Ig producing B cells. Some Ig production may persist, probably due to surviving long-lived plasma cells of host origin (34). As a reflection of normal ontogeny, IgM production will reconstitute relatively early and, on average, reaches normal levels within the first 6 months after HSCT. Similar to IgM, IgG generally reaches normal levels in the second half of the first year, whereas normalization of IgA levels may take up to 5 years after HSCT. IgG subclasses, on average, reach normal serum levels within 5 months (IgG1), 9 months (IgG3), or up to 2 years (IgG2 and IgG4). However, the time frame is highly variable and can be influenced by several factors such as the underlying disease, stem cell source, and type of donor (16, 31–33).

For complete humoral immune reconstitution after HSCT, generation of a diverse BCR repertoire is necessary. Literature on the diversification of the BCR after HSCT is limited. In the last 10 years, investigation of BCR diversification after HSCT has stagnated and studies performed are limited by older techniques or are difficult to generalize because of the small sample size. Analysis of the pattern of VH3- and VH4-gene usage based on 700 rearrangements in four patients suggested that the B-cell receptor repertoire shows the same (limited) repertoire of VH genes at 90 days and 1 year after HSCT (35). Other studies showed evidence that generation of the new repertoire occurs gradually and suggest that the CDR3 regions post HSCT are similar to CDR3 regions in adults and do not follow fetal ontogeny (36–38). The CDR3 length in the memory B cell compartment has a specific restriction compared to healthy controls, resulting in an oligoclonal repertoire early after HSCT (39). These methods only provide rough information about the BCR repertoire post HSCT. In adults, one study investigated the IGH repertoire before and after HSCT in acute myeloid leukemia patients using next generation sequencing. In general, they observed lower repertoire diversity after HSCT than before (40). Furthermore, each individual appeared to have highly unique and characteristic IGH repertoire of switched memory B-cells, which allowed the investigators to separate donor and recipient derived B cell clones. Interestingly, this showed in some cases persistence of recipient B cells which indicates that recipient B cells may still contribute to protective immunity after HSCT. The study analyzed the VH1 Ig repertoire, which represents only about 10% of all Ig sequences in humans. All of these studies exclusively investigated Ig heavy chain diversity. To the best of our knowledge, there are no studies investigating Ig light chain.

Based on available literature, HSCT-treated patients are frequently affected by an unbalanced, incomplete and, therefore, abnormal BCR repertoire, leading to impaired humoral immunity with associated risks of infectious and/or auto-immune complications. So far, studies have been limited by small cohorts and low-throughput or low resolution techniques. More in-depth analyses are needed to understand the integrated evolution of cellular and repertoire reconstitution and the influence of different HSCT-related factors on immune repertoire formation and B cell function require after transplantation.

## Factors Influencing B Cell Reconstitution

### Stem Cell Source

Although all different stem cell sources have curative potential, they differ in qualitative and quantitative characteristics of immune recovery and risk of specific HSCT-related complications such as graft vs. host disease (GVHD) (Table 1). Several studies suggest that umbilical cord blood (CB) is superior over bone marrow (BM) or peripheral blood stem cells (PBSC) with regard to time to B cell recovery and B cell differentiation (16, 41, 42). Total B cell counts, non-switched memory and switched memory B cells are higher in CB compared to BM and PBSC. In a mismatched donor setting, CB recipients show no significant better outcome for B cell reconstitution (62). The rapid reconstitution and better differentiation of B cells when using CB could be explained by the higher number of B lymphocyte progenitors in CB compared to BM (63). However, as precursor composition in matched and unmatched CB are the same, differences in CD19+ counts cannot be explained by higher progenitor numbers alone.

**Table 1:** Factors influencing B cell reconstitution.

**Factor**	**Influence on B cell reconstitution**	**References**
Stem cell source	CB: better B cell recovery (numbers) and B cell differentiation as compared to BM and PBSC. PBSC as compared to BM: higher B cell numbers in the early phase after HSCT (comparable numbers after 6 months)	(16, 33, 41–44)
Serotherapy	Ambiguous, some studies report delayed B cell reconstitution while others have indicated the opposite. Requires further study.	(16, 19, 31, 45–48)
MAC	Good humoral function, adequate B cell reconstitution and chimerism. BUT not always feasible.	(20, 39, 49–51)
RIC	Better survival in patients with pre-existing comorbidities or certain diseases, but not always optimal B cell reconstitution and IVIG dependence.	(52, 53)
TBI	Delayed B cell reconstitution.	(16, 19)
aGVHD	Significantly poorer B cell reconstitution, in both function and numbers. Higher grades of aGHVD seem to be associated with more extensively impaired humoral immunity.	(16, 54–57)
cGVHD	Poor B cell reconstitution mainly due to reduced numbers of B cell progenitors and unswitched memory B cells. Regulatory B cells (Bregs) are found to be reduced. Severity of cGVHD seems to correlate with the number of Bregs. cGVHD increases the frequency of activated B cells.	(16, 21, 33, 55, 58–61)

Between BM and PBSCs, differences in B cell reconstitution mainly exist in the early stage after HSCT. B cell numbers seem to recover faster when using PBSCs compared to BM (33, 43, 44). Still, with both stem cell sources, naïve, non-switched memory and switched memory B cells will remain below the normal values on the long term (16, 43). After 6 months, no differences are shown in B cell recovery using BM compared to PBSC (33, 43, 44).

Stromal cells and T cells are important for optimal B cell development and functionality in CB, BM and PBSC grafts (64–67). In CB more primitive and higher numbers of the stromal progenitors and primitive HSCs are reported compared to BM and PBSC (67). Cotransfer of stromal cells and T cells in the graft, as well as *ex vivo* modification of the grafts, such as CD34 selection, CD3/CD19 depletion and TCRα/β depletion could indirectly cause differences in functional and quantitative B cell reconstitution after HSCT.

### Serotherapy and Chemotherapeutic Conditioning

Serotherapy makes use of antilymphocyte antibodies which target T cells and other leukocyte populations, with the aim to reduce the risk of graft rejection and acute graft vs. host disease (aGVHD) (68, 69). The most prominent agents used are anti-thymocyte globulin (ATG) and alemtuzumab. Whereas ATG is a polyclonal immunoglobulin, alemtuzumab is a monoclonal antibody against CD52 (70). Both agents induce elimination of B cell populations (71, 72). A number of studies have reported on the impact of ATG on B cell reconstitution. Although some studies have reported a delay on immune reconstitution others have indicated the opposite (19, 31, 45–47). Whether these differences are explained by disease-specific characteristics, donor type, ATG exposure or the type of ATG is currently unresolved and requires further study. Similar to ATG, alemtuzumab has been reported to result in delayed B cell reconstitution. The kinetics of B cell reconstitution after alemtuzumab are variable and may as well be dependent on the same parameters as in case of ATG (16, 48).

Both in malignant and non-malignant diseases, achievement of full donor chimerism is often preferred to cure the primary disease and obtain stable graft function. In those cases myeloablative conditioning (MAC) is usually required. MAC often results in donor chimerism of all lineages and thereby in robust B cell reconstitution and function (20, 39, 49–51). However, in an increasing proportion of children a reduced intensity regimen is preferred either because the underlying disease or the pre-existing co-morbidities preclude a MAC approach. In these patients reduced intensity conditioning (RIC) is used, causing incomplete and reversible myelosuppression. Conditioning with RIC is associated with better survival, due to favorable toxicity profile and thus lower transplant-related mortality (52). However, RIC is associated with an increased incidence of partial/mixed chimerism and graft rejection which may result in suboptimal B cell function and the need for immunoglobulin supplementation (52, 53). Furthermore, the use of total body irradiation (TBI) is associated with delayed B cell immune reconstitution (16, 19). The mechanism behind TBI and the impaired reconstitution is not fully understood, but lower naïve B cells and switched memory B cells have been observed for up to 2 years.

### Graft vs. Host Disease

GVHD is a frequent complication of allogeneic HSCT which is responsible for significant transplant-related morbidity and mortality. aGVHD is mediated primarily by alloreactive donor T cells. The donor T cells are activated by host antigen presenting cells, which could be B cells. In general, GVHD is associated with significantly poorer B cell reconstitution, in both function and numbers (16, 54, 55). Higher grades of aGHVD seem to be associated with more extensively impaired humoral immunity, to which probably both GVHD itself as well as the associated immunosuppressive therapies contribute (56, 57). In chronic GVHD (cGVHD), poor B cell reconstitution seems mainly due to reduced numbers of B cell progenitors and unswitched memory B cells (16, 21, 33, 55, 58). In adults, regulatory B cells (Bregs) are found to be reduced in patients with cGVHD compared to no cGVHD and healthy controls. Severity of cGVHD seems to correlate with the number of Bregs, indicating a role for these B cell subset in cGVHD (59, 60). Using mass cytometry, it appeared that specific B cell subpopulations can be distinguished in patients suffering from different grades of cGVHD (61). Patients with severe cGVHD had an increased frequency of activated B cells, defined as CD38+ CD39+ CXCR5+ HLA-DR+ B-cells, compared to patients with moderate cGVHD. Furthermore, activated B cells were found at a reduced frequency in patients with mild cGVHD compared to patients without cGVHD (61). Regarding pathophysiology, whereas aGVHD is considered to be primarily mediated by T cells, an important role for donor B cells is assumed in the complex immune pathology of cGVHD (73–77). However, through antigen presentation, cytokine production and other immunoregulatory functions, it is hypothesized that B cells take part in the pathophysiology of all types of GHVD (78–82). Therefore, B cells have been targeted with several therapies such as Rituximab, Bortezomib, and Ibrutinib in both murine models and patients with GVHD, with promising clinical results (83–85).

## Infections and Vaccination

In a significant proportion of HSCT recipients, antibody titers of vaccine-preventable diseases decline over the years, if these recipients are not revaccinated (86–89). Vaccination responses after HSCT are dependent on both T- and B cell reconstitution. An exception is the polysaccharide antibody response, which is completely B cell dependent (90). The polysaccharide antibody response plays a role in the immunization against encapsulated bacteria, such as pneumococcus. HSCT recipients are more susceptible to infections during the post transplantation period (91–93). The risk of pneumococcal invasive disease is increased both early and late after HSCT, reaching 30-fold higher risks compared to the general population after 10 years, suggesting long lasting defects in B cell reconstitution even after revaccination (94–97). The risk of pneumococcal disease correlates with presence/occurrence of GVHD, suggesting a link between the functional dysregulation of B cells and GVHD (21, 94, 96). Whereas, an association with hypogammaglobulinemia has been reported, increased susceptibility to pneumococcal disease is most often due to a more selectively impaired immune response against polysaccharides (27, 28, 95). A poor response to polysaccharide vaccines, is hypothesized to be caused by the lack of unswitched memory B cells, which are reduced after transplantation (98). A poor response to polysaccharide vaccines is hypothesized to be caused by reduced MZ B cells or IgM memory B cells, which are reduced after transplantation (16, 17, 20, 21, 98). Reduced numbers of IgM memory B cells and increased risk of encapsulated bacterial infection has also been observed in young children, patients with common variable immune deficiency (CVID) and splenectomized patients (29, 99–101). Revaccination usually occurs with inactivated vaccines, as live attenuated vaccines have the potential to induce active disease in immunosuppressed patients. However below normal values, class switched memory B cells are observed as early as 3 months after HSCT (17). It is largely unknown if these cells are already capable of an immune response, taking into account the slow reconstitution of CD4+ T cells. In current guidelines, revaccination starts 3-6 months after HSCT, but looking at thresholds of the CD4+ T cells and ability for class switch recombination might be a useful biomarker to guide the timing of vaccination compared to fixed time point after HSCT. Live attenuated vaccines could be considered two years after HSCT, in patients without cGVHD or immunosuppression (102).

## Future Prospects

B cell reconstitution after HSCT including BCR repertoire formation and diversification, is awaiting new insights in order to develop better treatment strategies for prevention of clinical complications due to defects in B cell mediated immunity. For example, clinical outcome is still suboptimal in a proportion of HSCT-treated severe combined immunodeficiency (SCID) patients, due to humoral immune dysfunction. Some of these patients still suffer from persistent (humoral) immunodeficiency, auto-immunity and/or immune dysregulation leading to impaired quality of life (103). Although SCID represents the prototype of inherited immune disorders, an increasing spectrum of patients with inherited immune disorders is being genetically identified (>250 monogenetic diseases) (104). This steadily growing group of transplanted patients with non-SCID inherited immune disorders faces similar challenges regarding the long term quality of their (sometimes partially) corrected immune system. To evaluate the long term quality of immune reconstitution, B cell immunity recovery can serve as an indicator of immune fitness after HSCT.

We have limited information on the diversity of the BCR repertoire in the various B cell subsets in both peripheral blood and BM of HSCT-treated patients. Important insights have been obtained through flow cytometric analysis of peripheral B-cells after HSCT. However, to gain better understanding of numerical and functional B-cell reconstitution more in depth-analysis of cellular dynamics (using flow cytometry and KREC analysis), molecular aspects (BCR repertoire analysis) and antigen specificity is needed. Extensive analysis of B cell reconstitution can be done through modern flow cytometry and sequence techniques or even simultaneously with mass cytometry (105, 106). With modern sequencing techniques, it is possible to look at both the BCR heavy and light chain. Combined with single cell RNA sequencing, the integrated gene expression per cell within individual patients can be investigated (107). These modern techniques/methodology will make it possible to investigate the influence of aforementioned parameters on B cell repertoire development after HSCT in an innovative and more accurate way. This could revolutionize the knowledge about the B cell reconstitution and pinpoint the individual B cell maturation problems of transplanted patients.

## Author Contributions

NvdM and MvdB contributed conception of the paper; NvdM did literature search and wrote the first draft of the manuscript; DB, MvdB, and AL wrote sections of the manuscript. All authors contributed to manuscript revision, read and approved the submitted version.

### Conflict of Interest Statement

The authors declare that the research was conducted in the absence of any commercial or financial relationships that could be construed as a potential conflict of interest.
